# Opening label, dynamic prospective cohort study on the small focus less than 1.0 cm shown by type B ultrasound in breast

**DOI:** 10.1097/MD.0000000000020158

**Published:** 2020-05-08

**Authors:** Aiping Shi, Yi Dong, Xinpeng Xie, Haiying Du, Ming Yang, Tong Fu, Dong Song, Bing Han, Gang Zhao, Sijie Li, Ye Du, Hongyao Jia, Di Wu, Zhimin Fan

**Affiliations:** a Department of Breast Surgery, First Hospital of Bethune of Jilin University; b Department of Breast Surgery, Jilin Province Cancer Hospital, Changchun; c Department of Ultrasonic Diagnosis and Treatment Center, Jilin Provincial Central Hospital, Jilin, China.

**Keywords:** biopsy, breast ultrasound, follow-up, mammography, nonpalpable lesions

## Abstract

**Background:**

A consensus has not been achieved regarding the treatment of small nonpalpable breast lesions, and the purpose of this study was to prospectively investigate nonpalpable lesions less than 1.0 cm in diameter to explore the risk factors for such lesions and determine appropriate treatment of such kind of lesions.

**Methods:**

A total of 1039 patients with small lesions less than 1.0 cm in diameter who underwent mammography and ultrasound from 2009 to 2010 in our institution were prospectively enrolled. Among them, 80 patients underwent biopsy, whose lesions grew by more than 30% of its original size, with an unclear boundary or irregular shape. All patients were followed-up for an average of 24 months, and lesions identified as high-risk types, such as cancer or atypical hyperplasia, of tumors on pathological examination were labeled “meaningful lesions.” Then relevant factors affecting the detection of meaningful lesions were analyzed.

**Results:**

In total, 40 meaningful lesions including 2 breast cancers were detected, accounting for 3.8% and 0.2% of all patients, respectively. Univariate analysis identified smoking (*P* = .030), irregular shape (*P* = .018), unclear boundary (*P* = .024), and vascularization (*P* = .023) as risk factors for the detection of meaningful lesions (*P* < .05). On multivariate analysis, smoking and irregular shape were further identified as independent risk factors for the detection of meaningful lesions.

**Conclusion:**

The overall incidence of cancer among nonpalpable lesions with a diameter less than 1.0 cm is low. Biopsies are strongly recommended for patients who are smokers or who have small lesions with an irregular shape, whereas regular follow-up observation is likely safe for other patients with small, non-palpable breast lesions.

## 1 Introduction

Breast cancer is the most common malignancy in women, 249,000 cases of breast cancer being reported in China in 2011, with a corresponding incidence of 37.86/100,000 and mortality rate of 9.21/100,000.^[1]^ The incidence of breast cancer increases with age, female patients over 45 years old accounting approximately 70% of all breast cancer patients worldwide and 69.75% specifically in China.^[2]^ Early detection and diagnosis are particularly critical in the breast cancer treatment, and diagnosis by pathology is the gold standard^[3]^ Intraductal papilloma and sclerosing adenosis lesions are recognized as high risk lesions^[4]^ and their early diagnosis provides guidance for subsequent therapy. Even for breast cancer types that are not considered high-risk, such as fibroadenoma, an early definitive pathological diagnosis can reduce the anxiety of patients. However, biopsy of non-high-risk lesions such as ductal dilatation, adenosis, and lobular hyperplasia represents an unnecessary invasive procedure for patients. Therefore, the ability to identify meaningful lesions for which biopsy is needed would be of great benefit.^[5]^

At present, mammography and ultrasound are the most frequently imaging techniques used for breast lesion screening. The breast imaging-reporting and data system (BI-RADS) classification is widely applied in clinical practice.^[6]^ More than 98% of breast lesions categorized as BI-RADS classification 3 are benign, and follow-up yearly for 2 to 3 years is considered safe for these lesions.^[7,8]^ Unfortunately, research in Japan has shown that mammography has limited ability to detect lesions in women with small and dense glands, and the sensitivity of ultrasound for detecting small lesions is reduced to only 43%.^[9]^

From what has been discussed above, at present, breast cancer screening is mainly conducted by ultrasound and mammography, but the sensitivity of the both examinations is insufficient, so we can have to rely on other factors. In addition, no consensus has been reached regarding these factors related to these nonpalpable nodules. For the treatments of such lesions, surgery or follow-up, there is still no agreement. Therefore, a method for detecting high-risk lesions of breast cancer at an early stage while also minimizing unnecessary invasive operations is an urgent clinical need to reduce the mortality of breast cancer.

## 2 Material and methods

### 2.1 Study population

This prospective study was approved by the Ethics Committee of the First Hospital of Bethune of Jilin University (Reference Nos. 2009-016) under the project registration number CHiCTR-OCH-11001459 (http://www.chictr.org.cn). Bilateral breast were screened by gray-scale ultrasonography or mammography examination and vascularization was detected by color doppler. The inclusion criteria for the study were as follows: Age 18 or older; Patients admitted to the outpatients or inpatients of the first hospital of jilin university from 2009 to 2010; Lesion size less than or equal to 1.0 cm on breast ultrasound images or mammography; BI-RADS classification of 3 or lower by ultrasound or mammography according to the guidelines for breast cancer;^[7]^ Willingness to participate in the study and provide written informed consent. The exclusion criteria were: A request for discontinuation of follow-up; BI-RADS classification higher than 3 by ultrasound or mammography; Incomplete information; Previous history of malignant tumors; Complicated with other serious organ injuries and other diseases; Pregnant or breastfeeding women; Patients Participanting in other clinical trials;

### 2.2 Imaging-based diagnosis and evaluation

Each patient underwent physical examination by a clinician with 5 to 10 years of experience, and then imaging examinations were performed. Breast ultrasound was conducting using a KR-S80 ultrasound machine (Kyle Medical Electronics Co., Ltd, Xuzhou, China) to scan each quadrant of the breast:

(1) Color doppler ultrasound was used to screen breast cancer.
(2) Probe frequency:10 to 12 MHz.
(3) Apply coupling agent with probe and scan the breast horizontally and longitudinally.
(4) Observe lesion size, shape, boundary, calcification, and vascularization distribution.

Mammography examination was performed using an MCR-6000 mammography machine (McRae Electronics Co., Ltd, Shenzhen, China) in the craniocaudal and mediolateral oblique positions by the pressure fixation method. The color Doppler ultrasound and mammography images were evaluated by 2 radiologists with 5 to 10 years of experience. When the assessments of the 2 radiologists differed, senior radiologists made the final judgement.

### 2.3 Lesion observation and biopsy

All the enrolled patients were followed up by breast ultrasound every 6 months, and mammography was performed annually. If the size of a lesion remained unchanged or decreased, or if the lesion disappeared over four consecutive examinations, the patient was excluded from the study. All patients were followed up for at least 24 months (mean, 27 months; range, 24–30 months). Ultrasound-guided biopsy or minimally invasive surgery was performed if a lesion grew by more than 30% of its original size, for lesions with an unclear boundary and irregular shape.

### 2.4 Pathological evaluation of breast cancer lesions

Intraductal papilloma, sclerosing adenosis, radioactive scar, chronic inflammation of the breast, atypical hyperplasia, and breast fibroadenoma were defined as meaningful lesions. Non-high-risk lesions such as expansion of the duct, simple cyst, adenopathy, lobular hyperplasia and lesions without progression during long-term follow-up were defined as non-serious lesions requiring only follow-up observations.

### 2.5 Statistical analysis

The data were analyzed using SPSS (ver. 23.0, IBM Inc. Armonk, NY). The size of the small lesions was measured by color Doppler ultrasound. Data for multiple lesions are expressed in the form of mean ± standard deviation. The statistical analysis involved risk ratio analysis and chi-square test. If the theoretical frequency did not meet the relevant conditions, Fisher exact test was performed. Multivariate analysis used stepwise logistic regression, and all variables with a *P* value < .05 were included in the univariate analysis.

## 3 Results

**Figure 1:**
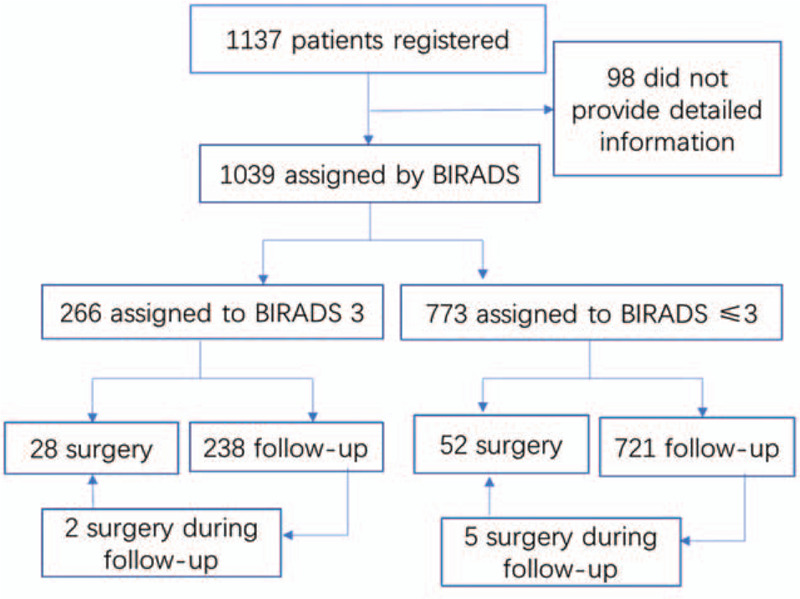
The numbers of surgery includes patients who underwent surgery directly and those who underwent surgery during follow-up.

**Figure 2:**
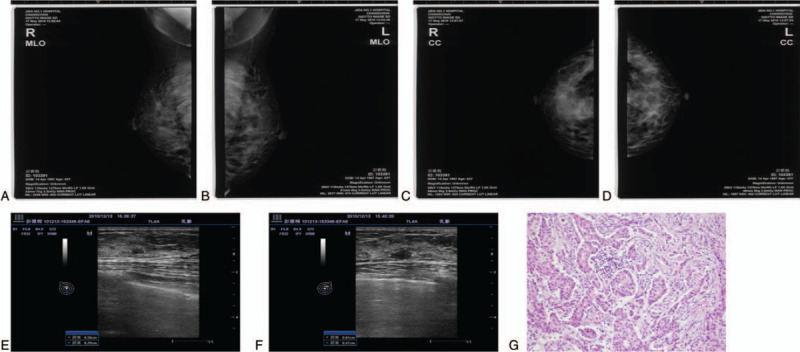
Examination results for a 36-yr-old female patient with a family history of breast cancer. (A-D) First mammography examination showing no obvious abnormal mass. (E-F) Color Doppler ultrasound images during follow-up at intervals of 3 mo showing that the tumor grew more than 30%. (G) Representative image from the pathologic examination, which confirmed the breast lesion as cancerous.

A total of 1137 female patients with an average age of 41.3 years (range, 35–75 years) were enrolled from January 2009 to December 2010. Among the 1137 patients enrolled in the study, a total of 98 patients (8.6%) were lost to follow-up, as shown in Figure 1. The sizes of nonpalpable lesions ranged from 0.30cm to1.00 cm. Of the 1039 patients who completed the study, 2 patients were diagnosed with cancer, for an incidence of 0.2%. Two hundred sixty-six patients (25.6%) had lesions of BI-RADS classification 3, and of these cases, 26 (9.8%) underwent surgery including 1 diagnosed with breast cancer and 240 patients chose follow-up observation. During the follow-up period, 2 patients underwent surgical treatment due to tumor enlargement by more than 30%, and no meaningful lesions were detected. Among the patients with lesions assigned to a BI-RADS classification lower than 3 only by mammography, 52 patients (6.7%) underwent mass biopsy. Follow-up observation was performed for 725 patients, of whom 5 patients underwent surgical treatment for tumor enlargement during follow-up and 1 patient received a pathological diagnosis of invasive breast cancer (Fig. 2). The results of postoperative and biopsy pathology examinations are shown in Table 1. Of the patients who underwent surgical treatment, pathologic examination confirmed the presence of meaningful lesions in 40 cases.

**Table 1:**
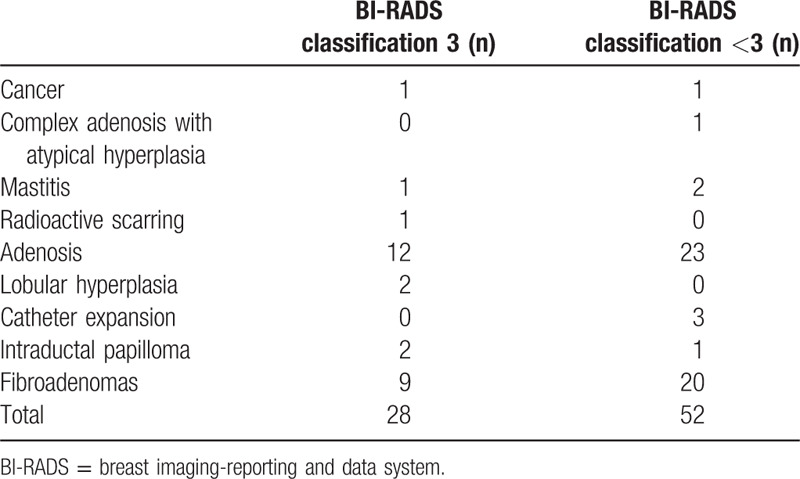
Summary of surgical pathology results.

Overall, among the 1039 patients, 40 patients were found to have meaningful lesions, and 999 patients had non-serious lesions for which only observation was recommended (Table 2). The percentages of patients over 45 years of age in these groups were 20% and 16.5%, respectively (*P* = .833). There was no significant difference in the detection rates of meaningful lesions between patients with or without a family history of breast cancer or pre- vs post-menopause (*P* = .403 and *P* = .185, respectively), nor did parity or menarche age have a significant effect on the detection of meaningful lesions (*P* = 1.000).

**Table 2:**
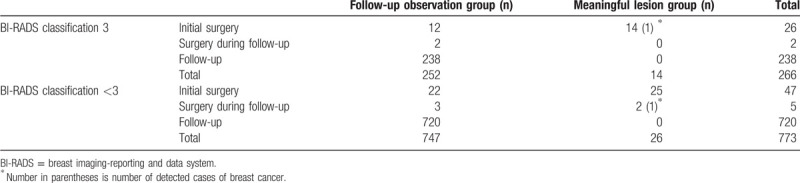
Detection of lesions in follow up observation group and meaningful lesions group.

Univariate analysis of all patients showed that smoking (*P* = .030), lesion shape (*P* = .018), lesion border (*P* = .024), and vascularization (*P* = .023) were significant risk factors for the detection of meaningful lesions (Table 3). On multivariate analysis, smoking (*P* = .021) and lesion shape (*P* = .007) remained significant factors influencing the detection of meaningful lesions (Table 4). The risk of meaningful lesions in smokers was 2.652 times showed in Table 5 that in non-smokers. The risk of meaningful lesions in patients with irregular lesions was 2.750 times (Table 5) that in patients with lesions of regular morphology. Other breast cancer-related factors, such as menopausal status, family history of breast cancer, and body mass index of 25 kg/m^2^ or greater, were not identified as statistically significant factors affecting the detection of meaningful lesions (*P* > .05).

**Table 3:**
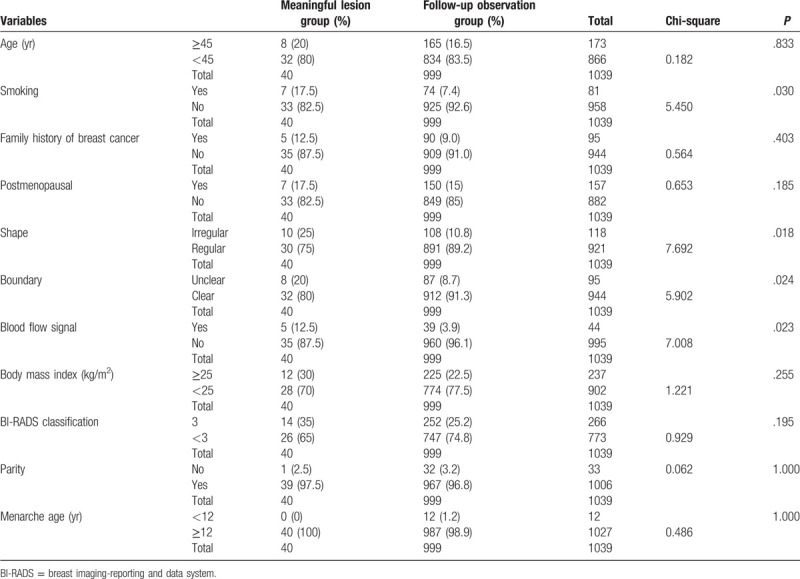
Patient characteristics.

**Table 4:**
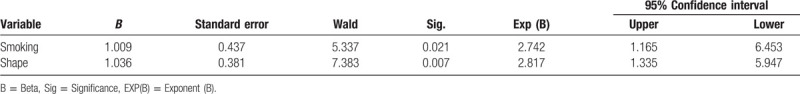
Assessment for each variable as a risk factor by multivariate analysis.

**Table 5:**
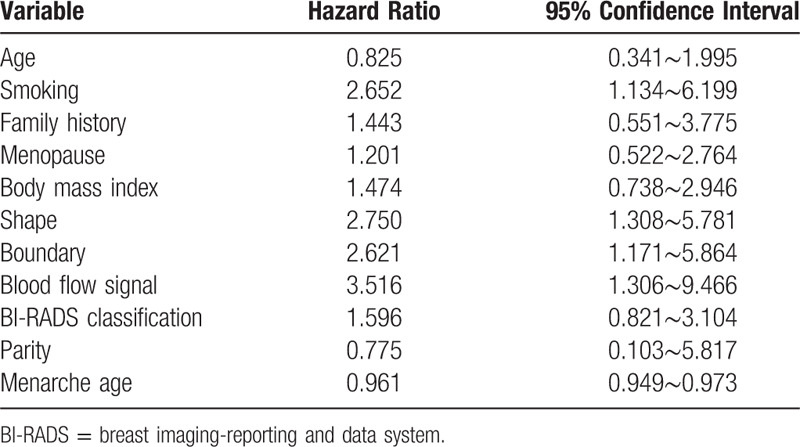
Assessment for each variable as a risk factor by univariate analysis.

## 4 Discussion

Nonpalpable mass is common lesion observed in routine clinical practice and in most of the cases is associated with a benign condition. However, some nonpalpable lesions may develop into malignant lesions, especially, if the shape of lesion is irregular and so on becomes necessary to exclude any malignant diseases. According to the study of Zhang et al,^[10]^ the sensitivity, specificity of color doppler ultrasound in the diagnosis of breast lesions were 84.3% and 83.5% respectively. And the sensitivity, specificity mammography in the diagnosis of breast lesions were 79.1% and 76.6% respectively. And then we use both examinations to assess the feature of breast lesions. In this study, we investigated patients’ mammography and ultrasound results as well as further biopsy findings to identify relevant factors for the detection of meaningful breast lesions, which might provide insight into appropriate treatment strategies for different types of lesions. Here we introduce the concept of “meaningful lesions” in reference to those that likely require more than follow-up observation. Our results demonstrated that smoking, vascularization, lesion shape, and lesion boundary were statistically significant factors for the detection of meaningful lesions confirmed by univariate analysis.

Smoking is a high-risk factor for breast cancer, and a study by Baglia et al^[11]^ showed that smoking can increase the risk of breast cancer by 24% compared with the risk among non-smokers. In our study, all smoking patients had a smoking history of more than 5 years and were currently active smoking, the detection rate of meaningful lesions was higher in smoking patients. Our findings are consistent with the results of the cohort study by Gaudet MM et al^[12]^ showed that non-smokers had a 24% lower risk of developing breast cancer than those who were smoking, while those with a history of smoking who had quit had a 13% higher risk of developing breast cancer than non-smokers.

On color Doppler ultrasound examination of the breast, a lesion with an irregular shape, unclear boundary, or vascularization is considered suggestive of the possibility of malignancy.^[13–15]^ In the present study, multivariate analysis showed that irregular shape was a significant imaging feature for the detection of meaningful lesions. Isidori et al^[16]^ performed color Doppler ultrasound breast examinations for nonpalpable lesions and reported that an irregular shape suggests the possibility of breast cancer. In the present study, univariate analysis showed that vascularization could indicate a meaningful lesion, but on multivariate analysis, this was no longer identified as a risk independent factor for meaningful lesions. At present, the use of vascularization in breast cancer screening is not clinically feasible anyway, and little is known about the relationship between nonpalpable lesions and vascularization. However, Madjar et al^[17]^ reported that vascularization contributed to the detection of breast cancer. Thus, additional research is needed to determine the significance of vascularization in the identification of meaningful breast lesions. Another feature affecting the diagnosis of breast cancer based on color Doppler ultrasound is the boundary of the lesion.^[18–21]^ In our study, univariate analysis identified an unclear boundary as a risk factor for the detection of meaningful lesions, and 20% of meaningful lesions detected had unclear boundaries.

The influence of other factors including age and family history on the detection of meaningful lesions were also investigated. Considering that the incidence of breast cancer is significantly increased in patients over 45 years of age, and less than 3% of breast cancer cases occur in women younger than 35 years,^[22]^ we analyzed patients over 35 years old and found that age did not affect the detection rate of meaningful lesions. Similarly, a family history of breast cancer did not statistically influence the detection of meaningful lesions in our study, nor was this factor reported to play a role in the early detection of meaningful lesions in previous studies.^[23–26]^

Alimoglu et al^[27]^ reported a detection rate of breast cancer among nonpalpable lesions of only 0.3% (2/562), which is consistent with our findings. Raza et al^[20]^ also investigated the biopsy findings for BI-RADS category 3 nonpalpable lesions and found that breast cancer is more common in lesions with significant morphological and size alterations. Therefore, if a patient has a history of smoking and breast ultrasonography reveals a lesion with an irregular shape, unclear boundary or abundant blood flow, a biopsy should be performed to determine the pathological type of the lesion. However, studies on the detection of meaningful lesions among small lesions remain limited, and multi-center studies in many regions of the world are still needed to characterize their occurrence, which will facilitate more reasonable and comprehensive diagnosis and treatment for patients with small lesions.

The present study has several limitations. First, the numbers of breast cancer cases and patients who underwent pathological biopsy were relatively small among the total population of 1039 patients. This may be because the patients in the cohort are not at high risk, and there may be selection bias in the population. Second, this was a single-center study, and multi-center studies are still needed to develop a more reasonable and comprehensive diagnosis and treatment strategy for small breast lesions.

## 5 Conclusion

Four main risk factors for the detection of meaningful breast lesions were identified, including smoking, vascularization, lesion shape, and lesion boundary. However, smoking and irregular shape were independent risk factors for the detection of meaningful lesions and both can indicate the detection of meaningful lesions. Therefore, surgical biopsy should be performed in patients who present with small lesions with an irregular shape and who are smokers. Otherwise patients with nonpalpable lesions who lack special requirements can be followed up regularly to avoid unnecessary invasive operations.

## Acknowledgments

We acknowledge Changgui Kou, School of Public Health of Jilin University, for provided statistical support for this study.

## Author contributions

Principal Investigator: Zhimin Fan.

Design, execution and quality control: Aiping Shi.

Case collection: Ming Yang, Song Dong, Tong Fu, Bing Han, Gang Zhao, Sijie Li, Ye Du, Hongyao Jia.

Follow-up: Yi Dong, Haiying Du.

Data processing: Di Wu.

Manuscript writing and data analysis: Xinpeng Xie.

Final approval of manuscript: all authors.
